# Over-expressions of *AMPK* subunits in ovarian carcinomas with significant clinical implications

**DOI:** 10.1186/1471-2407-12-357

**Published:** 2012-08-16

**Authors:** Cuilan Li, Vincent WS Liu, Pui M Chiu, David W Chan, Hextan YS Ngan

**Affiliations:** 1 Department of Obstetrics and Gynecology, LKS Faculty of Medicine, University of Hong Kong, Pokfulam, Hong Kong SAR, People’s Republic of China; 2 Department of Obstetrics and Gynecology, Queen Mary Hospital, 6th Floor, Professorial Block, Pokfulam, Hong Kong SAR, People’s Republic of China

**Keywords:** *AMPK*, *AMPK* subunits, Differential gene expression, Ovarian carcinoma

## Abstract

**Background:**

*AMP-activated protein kinase* (*AMPK*) has recently been considered as a potential target for cancer therapy. However, the expression status of various subunits of the heterotrimeric *AMPK* in human cancers is rarely reported. We decided to determine their expressions in ovarian carcinomas and their relationships with the disease.

**Methods:**

Expressions and locations of the *AMPK-α1*, *-α2*, *-β1*, *-β2*, *-γ1* and *-γ2* were detected by quantitative PCR (Q-PCR) and immunohistochemical staining (IHC). Their expression levels in ovarian tumors were compared with normal controls and also correlated with clinicopathological parameters.

**Results:**

Except *AMPK-α1*, expressions of the other five *AMPK* subunits are significantly higher in ovarian carcinomas as determined by Q-PCR. Although IHC detection of *AMPK-γ1* and *-γ2* were not successful, over-expressions of *AMPK-α2*, *-β1*, and *-β2* were further confirmed by IHC. Over-expressions of various *AMPK* subunits occurred independently and were mainly detected in the cytoplasm. Interestingly, *AMPK-α2* and *-β1* were also detected in the nucleus and cell membrane, respectively. Clinical correlation analyses indicate that expressions of different *AMPK* subunits are associated with different subtypes of carcinoma. High expression of *AMPK-α2* is significantly associated with endometrioid carcinomas. On the other hand, high expressions of *AMPK-β* and *-γ* subunits are associated with mucinous and serous carcinomas, respectively. Furthermore, high expressions of *AMPK-β1* and *-γ2* are also associated with early and late stages of disease, respectively. Finally, patients with high expression of *AMPK-α2* had better prognosis.

**Conclusions:**

Aberrant expressions of *AMPK* subunits may play important roles in ovarian carcinogenesis. Each *AMPK* subunit may have its own function other than just a component of the AMPK molecule. Correlations with clinical parameters suggest that expressions of *AMPK* subunits have different clinical implications in ovarian cancer development.

## Background

*AMP-activated protein kinase* (*AMPK*) is a well-known cellular energy-balancing sensor [1,2], an important cell proliferation inhibitor [3], and a potential target for cancer treatment [4]. Pharmacological activation of *AMPK* using AICAR or metformin can inhibit the growth or induce apoptosis of a wide spectrum of cancer cells, including cervical [5] and ovarian [6-8] cancers, through modulation of p53, p27, or p21 activities and cellular activities such as cell polarity and mitosis [2,3,9]

*AMPK* is a heterotrimer composed of a catalytic subunit (α) and two regulatory subunits (β and γ) [2]. Each subunit has different isoforms, namely, α1, α2, β1, β2, γ1, γ2, and γ3 encoded by distinct genes, which enable the yielding of 12 possible heterotrimeric combinations. The functional aspects of *AMPK* have been extensively studied recently and reviewed in metabolic diseases and human cancers [2,3]. In contrast, the expression levels of the individual subunits of AMPK and their clinical significance in human cancers are rarely investigated. It would be intriguing to delineate the expression status of all *AMPK* subunits in ovarian cancer and their potential correlations with clinical presentations.

Therefore, in the present study, quantitative PCR (Q-PCR) and immunohistochemical (IHC) staining were used to determine the expressions and cellular locations of six *AMPK* subunits (α1, α2, β1, β2, γ1, and γ2) in ovarian tissues. The expressions of *AMPK* subunits in ovarian cancer were then correlated with the clinical data.

## Methods

### Clinical samples

To recruit tissues for research study, patient’s consent was obtained before surgery at Queen Mary Hospital, Hong Kong. The use of the clinical specimens was approved by the local Institutional Review Board. Tumors containing more than 70% tumor cells were only used in the present study. A total of 76 tumor samples surgically resected from primary ovarian cancer patients and 53 normal ovary samples from benign diseases (including 5 borderline cases) were randomly selected for this study. The histological subtypes and disease stages of the tumors were classified according to International Federation of Gynecology and Obstetrics (FIGO) criteria. Clinical data of patients were retrieved from the records kept at the Department of Obstetrics & Gynecology, Queen Mary Hospital, Hong Kong.

### RNA extraction and quantitative PCR analysis

Total RNA from the ovarian tissues was prepared using the TRIzol reagent (Invitrogen). Quality and quantity of RNA were determined by agarose electrophoresis and spectrophotometry, respectively. The cDNA was then prepared using the Taqman reverse transcription kit according to the manufacturer’s instructions (Applied Biosystems, Foster City, CA). The gene expression assay kits were also purchased from Applied Biosystems. The primers and probes used to detect and quantify the expressions of *AMPK* subunits were *AMPK-α1* (Assay ID: Hs00178893_m1); *AMPK-α2* (Hs00178903_m1); *AMPK-β1* (Hs00272166_m1); *AMPK-β2* (Hs00271294_m1); *AMPK-γ1* (Hs00176952_m1); and *AMPK-γ2* (Hs00211903_m1).

The real-time quantitative PCR (Q-PCR) was performed in an ABI 7500 system (Applied Biosystems). The relative expression level of each gene was normalized against the endogenous *β-actin* (Product no: 4326315E) control. The *GAPDH* (Assay ID: Hs99999905_m1) control was also used to further verify the relative expression level of each gene. The relative quantity of the RNA expression was calculated using the comparative CT method with the 7500 System SDS software [10].

### Immunohistochemical staining

Immunohistochemical (IHC) staining for the six AMPK subunits was performed separately on an ovarian cancer tissue array (OVC1021) (Pantomics Inc, San Francisco, CA). The tissue array contains 97 cancer cases and 5 normal/benign cases. The antibodies against the six *AMPK* subunits were all purchased from Cell Signaling Technology, Inc., USA. They were anti-AMPK-α1 (Cat. no.: 2795); anti-AMPK-α2 (2757); anti-AMPK-β1 (4182); anti-AMPK-β2 (4148); anti-AMPK-γ1 (4187); and anti-AMPK-γ2 (2536). In addition, the anti-phospho-AMPK-α (2535) was also use to detect the phospho-AMPK-α. The details of the IHC staining procedures have been published elsewhere [11,12]. The percentages of positively stained cells in tumors and normal epithelia were assessed. The proportions of positive cells were ranged from 10 to 100%, while the intensity of staining was scored as 0 (negative), 1 (very weak), 2 (weak), 3 (moderate), 4 (intense), and 5 (very intense) in the most strongly stained tumor area. The immunoreactivity score for each case was taken as percentage of positive cells multiplied by the intensity of staining.

### Confocal microscopy

The cellular locations of *AMPK-α2*, *-β1* and *-γ2* were examined in the ovarian cancer cells, A2780CP and SKOV3. The pCMV6–AMPK-α2–GFP, pCMV6–AMPK-β1–GFP and pCMV6–AMPK-γ2–GFP tagged plasmids (OriGene Technologies) were used. The analytical procedure has already been reported in a previous study [12]. The fluorescence signals were captured through confocal microscopy.

### Statistical analysis

Statistical analysis was performed using Chi-squared test to evaluate the relationship between gene expression (low/high) and clinicopathological parameters, including age, grade, stage, histological subtype and recurrence. The nonparametric Mann–Whitney test was used to determine the significance on the difference in the distribution of gene expression in cancer, borderline and normal samples. Kaplan-Meier and the log rank test were used for survival analyses (both overall survival; from diagnosis to death of any cause and disease-free survival; from diagnosis to relapse). The SPSS 15.0 software was used to carry out the statistical analyses. All *P* values reported were two-sided and *P* < 0.05 was considered as statistically significant.

## Results

### Measurement of the mRNA levels of *AMPK* subunits in ovarian tissues

Ovarian tissues, including tumor and normal tissues, were used in the current study. The relative levels of the six *AMPK* subunits including α1, α2, β1, β2, γ1, and γ2 in ovarian tissues were measured using real-time Q-PCR. Expression of the *AMPK-γ3* subunit was not conducted because it is not expressed in ovarian tissues.

**Figure 1:**
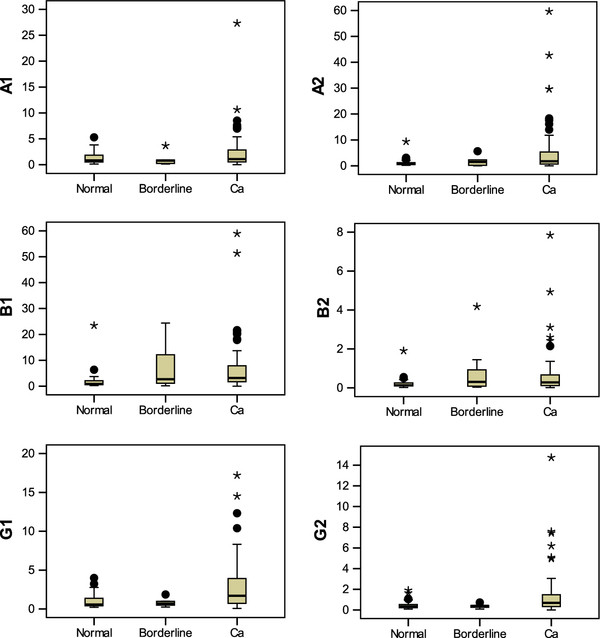
**Quantitative PCR analyses of the relative levels of six AMPK subunits in ovarian tissues.** Except for AMPK-α1, the boxplots indicate that statistically significant over-expressions of the other five AMPK subunits were detected in ovarian carcinomas compared with the normal. Borderline tumors were excluded from the analysis because of the small sample size. The abbreviations are A1 = AMPK-α1; A2 = AMPK-α2; B1 = AMPK-β1; B2 = AMPK-β2; G1 = AMPK-γ1; G2 = AMPK-γ2; Ca = Cancer; Normal N = 48; Borderline N = 5; Cancer N = 89.

The mRNA levels of all *AMPK* subunits, except *AMPK-α1*, were statistically significantly higher in ovarian carcinomas than those of the normal controls (Figure 1 & Table 1). Over-expressions of different *AMPK* subunits independently occurred and were not associated with one another.

**Table 1:** The medians and ranges of the expression levels of AMPK subunits in ovarian tissues

**AMPK subunit**	**Normal**	**Borderline**	**Cancer**	***P*value**
α1	Median = 0.847	0.810	1.091	0.320
	Range = 5.15	3.554	27.317	
α2	Median = 0.766	1.525	1.791	**<0.001**
	Range = 9.205	5.652	59.627	
β1	Median = 0.873	2.72	3.189	**<0.001**
	Range = 6.134	24.258	58.943	
β2	Median = 0.132	0.311	0.283	**0.001**
	Range = 0.53	4.14	7.83	
γ1	Median = 0.568	0.706	1.693	**<0.001**
	Range = 3.76	1.63	17.14	
γ2	Median = 0.341	0.39	0.685	**0.001**
	Range = 1.813	0.641	14.732	

### Immunohistochemical analyses of the protein levels and cellular locations of *AMPK* subunits in ovarian tissues

To further confirm the over-expressions of *AMPK* subunits in ovarian carcinomas, IHC staining of each *AMPK* subunit was carried out separately on an ovarian tissue array slide.

**Figure 2:**
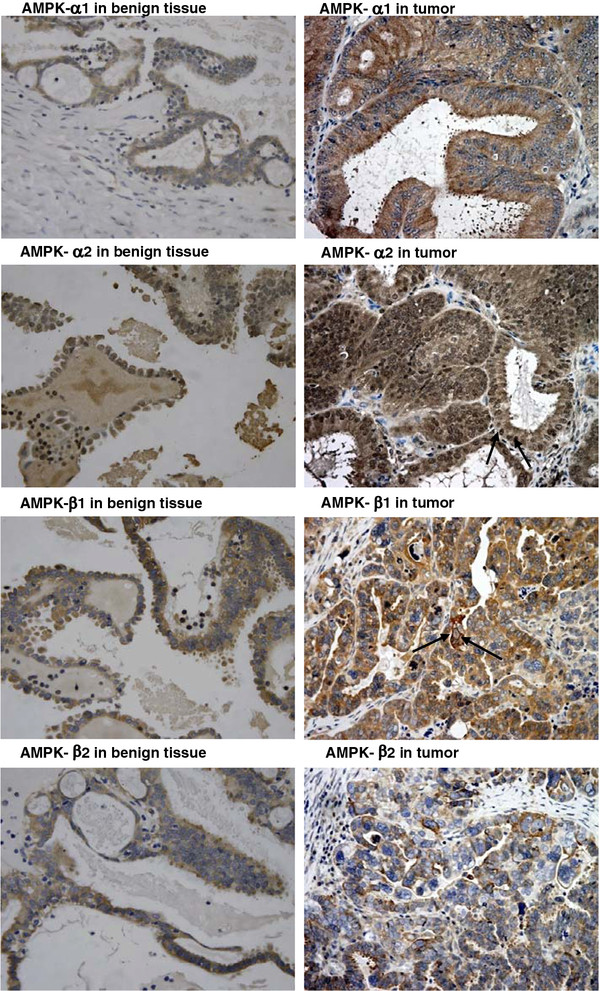
**Immunohistochemical staining of AMPK-α1, -α2, -β1, and -β2 expressions in ovarian tissues.** The over-expression of AMPK-α2 was observed in this representative tumor case. The expression of AMPK-α1 was detected in the cytoplasm only, whereas the expression of AMPK-α2 was detected in both cytoplasm and nucleus (indicated by an arrow). N.B. For AMPK-α1, in this particular tumor sample, AMPK-α1 expression was higher than the normal. This clearly indicated that AMPK-α1 was only expressed in the cytoplasm. Over-expressions of AMPK-β1 and AMPK-β2 were also observed in the two representative tumor cases. Expression of AMPK-β2 was detected in the cytoplasm only, whereas expression of AMPK-β1 was detected in both cytoplasm and cell membrane (indicated by an arrow).

IHC was successful using the antibodies against *AMPK-α1*, *-α2*, *-β1*, and *-β2*. However, IHC using antibodies against *AMPK-γ1* and *-γ2* subunits was not successful, and the protein levels of the two γ subunits cannot be determined. Consistent with Q-PCR results, *AMPK-α2*, *-β1*, and *-β2* subunits were over-expressed in ovarian carcinomas compared with the normal controls (Figure 2), whereas *AMPK-α1* expression generally had no significant difference between cancer samples and normal controls.

**Figure 3:**
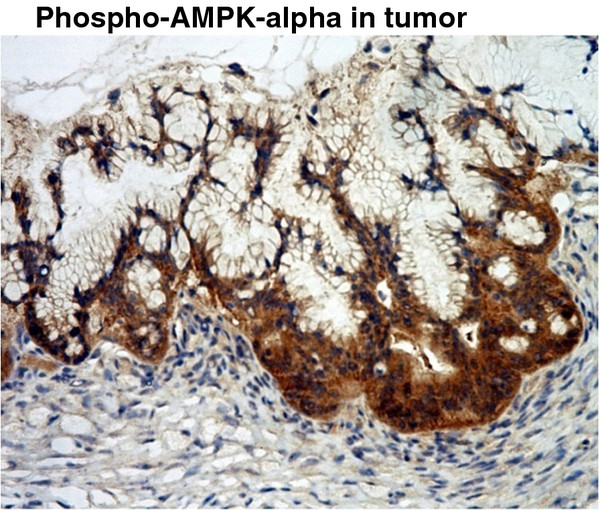
**Immunohistochemical staining of pAMPK-α expression in ovarian tissue.** The active form of AMPK, pAMPK-α, was only detected in the cytoplasm.

Concerning the cellular locations of the *AMPK* subunits, all four subunits were detected in the cytoplasm. In addition, *AMPK-α2* and *-β1* were also detected in the nucleus and cell membrane, respectively (Figure 2). The active form of AMPK, the phospho-AMPK-α was only detected in the cytoplasm (Figure 3).

### Cellular locations of *AMPK* subunits in ovarian cancer cells

**Figure 4:**
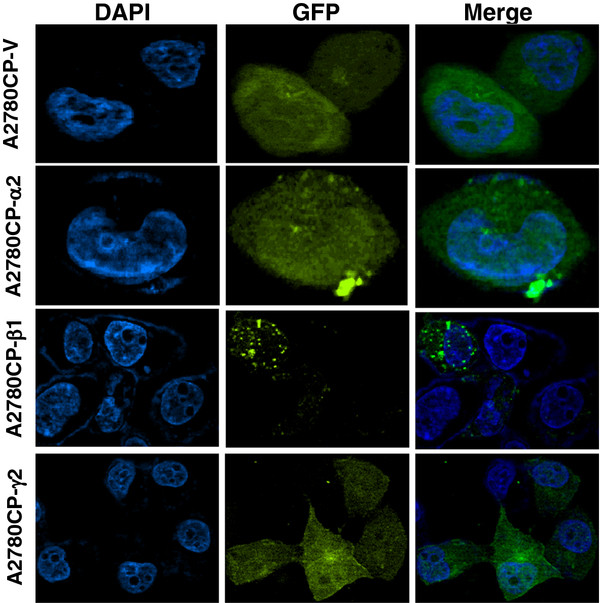
**Localization of AMPK subunits in A2780CP cell.** Enforced expressions of AMPK-α2, -β1, and -γ2 tagged with GFP were carried out in A2780CP. The expression of AMPK-α2 was evenly detected in both cytoplasm and nucleus. Expression of AMPK-β1 was observed in the cytoplasm as well as in the cellular membrane. The expression of AMPK-γ2 was evenly detected in the cytoplasm, membrane, and nucleus in A2780CP cell. A2780CP-V is a GFP vector control.

To confirm further the cellular locations of the *AMPK* subunits detected in IHC, confocal microscopy was used to localize the *AMPK* subunits in ovarian cancer cells with separately enforced expression of the subunits in A2780CP cell (Figure 4). Expression of *AMPK-α2* was detected in both cytoplasm and nucleus. Expression of *AMPK-β1* was observed in the cytoplasm as well as in the cellular membrane. Thus, the expressions of *AMPK-α2* and *AMPK-β1* detected either by IHC or confocal microscopy match with each other. Although IHC was not successful in detecting *AMPK-γ2*, expressions of *AMPK-γ2* were evenly detected in the cytoplasm, membrane, and nucleus of the A2780CP cell (Figure 4). The cellular locations of *AMPK-β1* and *-γ2* in another ovarian cancer cell SKOV3 were shown in Additional file 1: Figure S1. These results indicate that the expressions of AMPK subunits are quite distinct from one another.

### Differential expressions of *AMPK* subunits in different histological subtypes of ovarian carcinoma

After determining the expression levels of the various *AMPK* subunits by Q-PCR, the relationships between the expression levels of the various *AMPK* subunits and clinicopathological data were analyzed. The ovarian carcinoma samples were divided into two groups based on the relative expression levels of the *AMPK* subunits, that is, greater than or smaller than/equal to two-folds of the mean of the relative expression levels of the normal samples. The two groups of samples were then correlated with the clinicopathological parameters of the samples used in the current study. Table 2 summarizes the results of all correlations. The details of the correlation analyses are shown in Additional file 2: Table S1 to Table S7.

**Table 2:** Summary of association between high expression of AMPK subunits and clinical data

**AMPK subunit**	**Chromosomal location**	**Cell type**	**Grade**	**Stage**	**Recurrence**
Alpha 1	5p13	Mucinous^*^	NC	NC	NC
Alpha 2	1p32	Endometrioid	NC	NC	Lower rate
Beta 1	12q24	Mucinous	NC	Early	NC
Beta 2	1q21	Mucinous	NC	NC	NC
Gamma 1	12q13	Serous	NC	NC	NC
Gamma 2	7q36	Serous	Low	Late	NC

^*^Although AMPK-α1 is not generally significantly over-expressed in ovarian cancer, high expression of the AMPK-α1 still has an association with mucinous carcinoma (Additional file 2: Table S1). NC = No Correlation.

The high expression of the *AMPK-α2* subunit was found to be associated with endometrioid carcinomas (Additional file 2: Table S2). On the other hand, the high expressions of the *AMPK-β* and *-γ* subunits were found to be associated with mucinous (Additional file 2: Table S3 and Table S4) and serous carcinomas (Additional file 2: Table S5 and Table S6), respectively. Clear cell carcinomas had no association with any of the high expressions of the AMPK subunits (Additional file 2: Table S1 to Table S6). Since the antibodies for detecting AMPK-γ subunits were not good enough for IHC, we can only present the IHC staining quantifications of the AMPK-α and β subunits. The results are shown in Additional file 2: Table S8-Table S11. However, since the numbers of samples of the various histological subtypes in the tissue various greatly, it is not easy to match the results between Q-PCR and IHC staining. Nevertheless, the results determined by Q-PCR and IHC staining match one another in the grade and stage of diseases as described below.

### Expressions of AMPK subunits in different stages of ovarian carcinomas

The expressions of various *AMPK* subunits were also correlated with the stages of the disease. High expressions of *AMPK-β1* (Additional file 2: Table S3) and *AMPK-γ2* (Additional file 2: Table S6) were found in the early and late stages of the disease, respectively. The expressions of other *AMPK* subunits had no correlation with the stages of the disease. The correlation between *AMPK-β1* expression and the stage of the disease was further confirmed by IHC results (Additional file 2: Table S10).

### Correlation between AMPK-γ2 and grades of ovarian carcinomas

The high expression of *AMPK-γ2* was associated with low-grade tumors (Additional file 2: Table S6). However, this result cannot be further confirmed because IHC staining of *AMPK-γ* subunits was not successful. The expressions of other *AMPK* subunits had no association with the grades of tumors.

### Correlation between AMPK-α2 and disease survival

**Figure 5:**
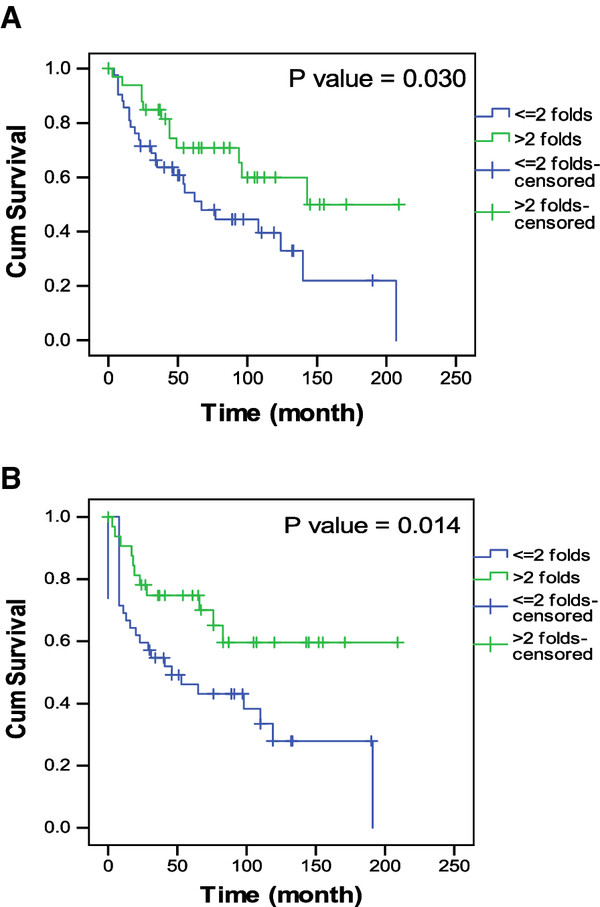
**Patients’ survival analyses.** Patients with high expression of AMPK-α2 had (**A**) better overall survival and (**B**) disease free survival.

Patients with high expressions of *AMPK-α2* had less disease recurrence rate (Additional file 2: Table S2) and better overall and disease-free survival rate (Figure 5). This finding is consistent with the results described in another report [13].

## Discussion

In the present study, the detection of the over-expressions of *AMPK* subunits in ovarian carcinomas was described. The high expressions of the *AMPK* subunits have significant correlations with one or more clinicopathological parameters. Concerning *AMPK-γ* subunits, the antibodies from another source were also used. However, they were still not good enough to reveal the expression of *AMPK-γ* subunits at the protein level by IHC. From Western blotting analysis, AMPK -γ1 and -γ2 were detectable in a number of human ovarian surface epithelial cell lines and ovarian cancer cell lines (data not shown). Thus, we excluded the possibility of negative expression of AMPK -γ1 and -γ2. Nevertheless, based on the good correlation between Q-PCR and IHC results on *AMPK-α* and *-β* subunits, the expressions of the *AMPK-γ* subunits measured by Q-PCR may also reflect the actual protein levels of the *AMPK-γ* subunits in ovarian tissues. Certainly, further confirmation is needed when antibodies against *AMPK-γ* subunits with the quality for IHC analysis become available in the future.

*AMPK* is a heterotrimer containing the α, β, and γ subunits, all of which are essential for kinase activity [2]. Thus, the expressions of the three different subunits should be regulated closely to keep their levels in balance for the formation of the *AMPK* complex. However, *AMPK-α2*, *-β1*, *-β2*, *-γ1*, and *-γ2* were independently over-expressed in ovarian carcinomas. *AMPK* subunits are encoded by distinct genes located at different chromosomal regions (Table 2). This may explain their independent over-expressions during ovarian carcinogenesis. Further, the association between the expressions of a particular *AMPK* subunit with a particular histological subtype of ovarian carcinoma further demonstrated that *AMPK* subunits can express independently to one another. Our data provide evidence on the complexity of genetic alterations during the development of cancer.

In a previous study, *AMPK-α1* was over-expressed in about 50% of cases of cervical cancer [11] but without association with any clinical parameters. In contrast, although no statistically significant over-expression of *AMPK-α1* was found in ovarian cancers, the high expression of *AMPK-α1* was associated with the mucinous type of ovarian carcinoma (Additional file 2: Table S1). The expression of *AMPK-α1* was predominantly found in the nuclei of cervical cancer cells [11]. In contrast, in the present study, *AMPK-α1* expression was mainly found in the cytoplasm of ovarian cancer cells. Based on the results of these two studies, the over-expressions of different *AMPK* subunits in human cancers can be hypothesized to be tumor-type specific. Different cancers may have different expression statuses of various *AMPK* subunits. Further investigation is needed to delineate the expression status of *AMPK* subunits in various human cancers and the clinical implications of their expressions during cancer development.

Consistent with another report [13], ovarian cancer patients with high expressions of *AMPK-α2* with better prognosis were also found in the present study. Tumors with low expressions of *AMPK-α2* may be more aggressive in nature. This observation leads to another important question on the potential functional roles of *AMPK* subunits in ovarian cancer. In addition, the IHC results indicated that *AMPK-α2* and *-β1* were also specifically detected in the nucleus and cell membrane, respectively. The active form of *AMPK* was only detected in the cytoplasm (Figure 3), so this result implied that *AMPK-α2* and *-β1* subunits may have distinct functions other than just being components of the heterotrimeric *AMPK* complex.

In an earlier study [14], under hypoxic condition, *AMPK-α2* expression was up-regulated and shuttled to the nucleus to promote cell survival by enhancing the hypoxia-induced *VEGF* expression in human glioblastoma cells. The potential function of *AMPK-α* subunits was further demonstrated. In a yeast two-hybrid experiment utilizing the HeLa cDNA library, the specific interaction between *AMPK-α* and *p73α* was shown. Both *AMPK-α1* and *-α2* can repress the tumor suppressor function of *p73α* and enhance the growth of H1299 (non-small cell lung carcinoma cell line) and U2OS (human osteosarcoma cell line) cells without involving the activity of AMPK [15]. In another study by Li et al. [16], the forced expression of *AMPK-β1* inhibits the growth of H1299 and U2OS cells. This finding indicates that different *AMPK* subunits have different functional roles in controlling cell growth. Knowledge on the functional roles of *AMPK* subunits in human cancers is still limited. In addition to expression status, further investigation of the functions of *AMPK* subunits is warranted. Functional studies may help in understanding the roles of these subunits in ovarian carcinogenesis.

## Conclusions

In this study, aberrant expressions of *AMPK* subunits were detected in ovarian carcinomas. More importantly, the high expressions of individual *AMPK* subunits have significant correlations with one or more clinicopathological parameters. These results suggest that expressions of *AMPK* subunits may play significant roles in the development of ovarian cancer.

## Competing interests

The authors declare that they have no competing interests.

## Authors’ contributions

CL and VWSL carried out most of the experiments and did the preparation of the manuscript. PMC did the immunohistochemical staining. HYSN provided the clinical samples and data and patient’s consent for the study. DWC and HYSN helped to analyze all the data and critical comments on the manuscript. All authors read and approved the manuscript to be submitted for publication.

## Pre-publication history

The pre-publication history for this paper can be accessed here:

http://www.biomedcentral.com/1471-2407/12/357/prepub

## Supplementary Material

Additional file 1 **Figure S1.** Localization of AMPK subunits in SKOV3 cell. Enforced expressions of AMPK-β1, and -γ2 tagged with GFP were carried out in SKOV3 cell. The expression of AMPK-β1 was observed in the cytoplasm as well as in the cellular membrane. The expression of AMPK-γ2 was evenly detected in the cytoplasm, membrane and, nucleus in SKOV3 cell. SKOV3-V is a GFP vector control. Click here for file

Additional file 2 **Table S1.** AMPK-α1 expression and clinicopathologic factors in ovarian cancer determined by Q-PCR. Table S2. AMPK-α2 expression and clinicopathologic factors in ovarian cancer determined by Q-PCR. Table S3. AMPK-β1 expression and clinicopathologic factors in ovarian cancer determined by Q-PCR. Table S4. AMPK-β2 expression and clinicopathologic factors in ovarian cancer determined by Q-PCR. Table S5. AMPK-γ1 expression and clinicopathologic factors in ovarian cancer determined by Q-PCR. Table S6. AMPK-γ2 expression and clinicopathologic factors in ovarian cancer determined by Q-PCR. Table S7. Frequencies of high and low expressions of AMPK subunits in various histological subtypes of ovarian cancer. Table S8. AMPK-α1 expression and clinicopathologic factors in ovarian cancer determined by IHC staining. Table S9. AMPK-α2 expression and clinicopathologic factors in ovarian cancer determined by IHC staining. Table S10. AMPK-β1 expression and clinicopathologic factors in ovarian cancer determined by IHC staining. Table S11. AMPK-β2 expression and clinicopathologic factors in ovarian cancer determined by IHC staining. Click here for file
