# Supplementary figures and images for: Over-expressions of AMPK subunits in ovarian carcinomas with significant clinical implications
Source: BMC Cancer. 2012 Aug 16;12:357. doi: 10.1186/1471-2407-12-357 (PMC3518102; doi:10.1186/1471-2407-12-357)

## Slide 1
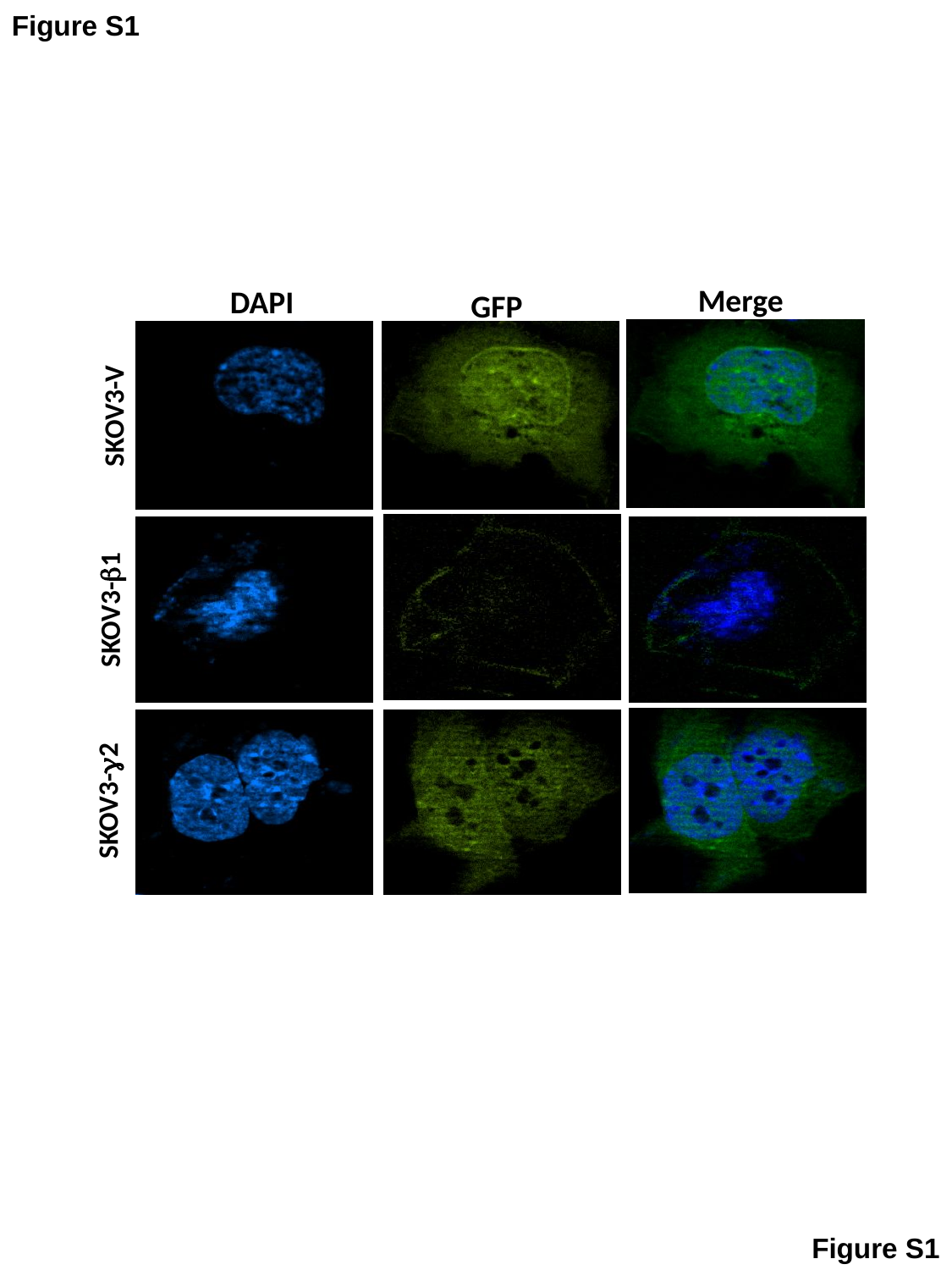

Figure S1
Merge
DAPI
GFP
SKOV3-V
SKOV3-β1
SKOV3-γ2
Figure S1

Supplement: Additional file 1 — Figure S1. Localization of AMPK subunits in SKOV3 cell. Enforced expressions of AMPK-β1, and -γ2 tagged with GFP were carried out in SKOV3 cell. The expression of AMPK-β1 was observed in the cytoplasm as well as in the cellular membrane. The expression of AMPK-γ2 was evenly detected in the cytoplasm, membrane and, nucleus in SKOV3 cell. SKOV3-V is a GFP vector control. [file 1471-2407-12-357-S1.ppt]
