# Supplementary material for: Over-expressions of AMPK subunits in ovarian carcinomas with significant clinical implications
Source: BMC Cancer. 2012 Aug 16;12:357. doi: 10.1186/1471-2407-12-357 (PMC3518102; doi:10.1186/1471-2407-12-357)
Supplement: Additional file 2 — Table S1. AMPK-α1 expression and clinicopathologic factors in ovarian cancer determined by Q-PCR. Table S2. AMPK-α2 expression and clinicopathologic factors in ovarian cancer determined by Q-PCR. Table S3. AMPK-β1 expression and clinicopathologic factors in ovarian cancer determined by Q-PCR. Table S4. AMPK-β2 expression and clinicopathologic factors in ovarian cancer determined by Q-PCR. Table S5. AMPK-γ1 expression and clinicopathologic factors in ovarian cancer determined by Q-PCR. Table S6. AMPK-γ2 expression and clinicopathologic factors in ovarian cancer determined by Q-PCR. Table S7. Frequencies of high and low expressions of AMPK subunits in various histological subtypes of ovarian cancer. Table S8. AMPK-α1 expression and clinicopathologic factors in ovarian cancer determined by IHC staining. Table S9. AMPK-α2 expression and clinicopathologic factors in ovarian cancer determined by IHC staining. Table S10. AMPK-β1 expression and clinicopathologic factors in ovarian cancer determined by IHC staining. Table S11. AMPK-β2 expression and clinicopathologic factors in ovarian cancer determined by IHC staining. [file 1471-2407-12-357-S2.doc]

**Table S1**

**AMPK-α1 expression and clinicopathologic factors in ovarian cancer determined by Q-PCR**

| Characteristics | | Total | Expression | |  |
| --- | --- | --- | --- | --- | --- |
|  |  |  | <=2.0 folds | >2.0 folds | *P* |
| Age (y) | <55 | 50 | 38 (76.0%) | 12 (24.0%) | 0.525 |
|  | >=55 | 26 | 18 (69.2%) | 8 (30.8%) |  |
| Grade | 1&2 | 26 | 16 (61.5%) | 10 (38.5%) | 0.110 |
|  | 3 | 31 | 25 (80.6%) | 6 (19.4%) |  |
| Stage | Early | 25 | 19 (76.0%) | 6 (24.0%) | 0.927 |
|  | Late | 49 | 36 (73.5%) | 13 (26.5%) |  |
| Cell types | Endometrioid | 22 | 19 (86.4%) | 3 (13.6%) | 0.109 |
|  | Others | 54 | 37 (68.5%) | 17 (31.5%) |  |
| Cell types | Mucinous | 18 | 8 (44.4%) | 10 (55.6%) | **0.001** |
|  | Others | 58 | 48 (82.8%) | 10 (17.2%) |  |
| Cell types | Serous | 25 | 17 (68.0%) | 8 (32.0%) | 0.525 |
|  | Others | 51 | 38 (74.5%) | 13 (25.5%) |  |
| Cell types | Clear cell | 16 | 13 (81.2%) | 3 (18.8%) | 0.439 |
|  | Others | 60 | 43 (71.7%) | 17 (28.3%) |  |
| Recurrence | + | 39 | 28 (71.8%) | 11 (28.2%) | 0.704 |
|  | - | 33 | 25 (75.8%) | 8 (24.2%) |  |

**Table S2**

**AMPK-α2 expression and clinicopathologic factors in ovarian cancer determined by Q-PCR**

| Characteristics | | Total | Expression | |  |
| --- | --- | --- | --- | --- | --- |
|  |  |  | <=2.0 folds | >2.0 folds | *P* |
| Age (y) | <55 | 50 | 25 (50.5%) | 25 (50.5%) | 0.201 |
|  | >=55 | 26 | 17 (65.4%) | 9 (34.6%) |  |
| Grade | 1&2 | 26 | 11 (42.3%) | 15 (57.7%) | 0.153 |
|  | 3 | 31 | 19 (61.3%) | 12 (38.7%) |  |
| Stage | Early | 25 | 13 (52.0%) | 12 (48.0%) | 0.517 |
|  | Late | 49 | 28 (57.1%) | 21 (42.9%) |  |
| Cell types | Endometrioid | 22 | 7 (31.8%) | 15 (68.2%) | **0.009** |
|  | Others | 54 | 35 (64.8%) | 19 (35.2%) |  |
| Cell types | Mucinous | 18 | 8 (44.4%) | 10 (55.6%) | 0.291 |
|  | Others | 58 | 34 (58.6%) | 24 (41.4%) |  |
| Cell types | Serous | 25 | 14 (56.0%) | 11 (44.0%) | 0.759 |
|  | Others | 51 | 27 (52.9%) | 24 (47.1%) |  |
| Cell types | Clear cell | 16 | 13 (81.2%) | 3 (18.8%) | **0.019** |
|  | Others | 60 | 29 (48.3%) | 31 (51.7%) |  |
| Recurrence | + | 39 | 27 (69.2%) | 12 (30.8%) | **0.022** |
|  | - | 33 | 14 (42.4%) | 19 (57.6%) |  |

**Table S3**

**AMPK-β1 expression and clinicopathologic factors in ovarian cancer determined by Q-PCR**

| Characteristics | | Total | Expression | |  |
| --- | --- | --- | --- | --- | --- |
|  |  |  | <=2.0 folds | >2.0 folds | *P* |
| Age (y) | <55 | 50 | 25 (50.0%) | 25 (50.0%) | 0.489 |
|  | >=55 | 26 | 15 (57.7%) | 11 (42.3%) |  |
| Grade | 1&2 | 26 | 11 (42.3%) | 15 (57.7%) | 0.483 |
|  | 3 | 31 | 15 (48.4%) | 16 (51.6%) |  |
| Stage | Early | 25 | 8 (32.0%) | 17 (68.0%) | **0.023** |
|  | Late | 49 | 30 (61.2%) | 19 (38.8%) |  |
| Cell types | Endometrioid | 22 | 12 (54.5%) | 10 (45.5%) | 0.770 |
|  | Others | 54 | 27 (50.0%) | 27 (50.0%) |  |
| Cell types | Mucinous | 18 | 4 (22.2%) | 14 (77.8%) | **0.005** |
|  | Others | 58 | 35 (60.3%) | 23 (39.7%) |  |
| Cell types | Serous | 25 | 13 (52.0%) | 12 (48.0%) | 0.990 |
|  | Others | 51 | 26 (51.0%) | 25 (49.0%) |  |
| Cell types | Clear cell | 16 | 7 (43.8%) | 9 (56.2%) | 0.318 |
|  | Others | 60 | 33 (55.0%) | 27 (45.0%) |  |
| Recurrence | + | 39 | 22 (56.4%) | 17 (43.6%) | 0.830 |
|  | - | 33 | 17 (51.5%) | 16 (48.5%) |  |

**Table S4**

**AMPK-β2 expression and clinicopathologic factors in ovarian cancer determined by Q-PCR**

| Characteristics | | Total | Expression | |  |
| --- | --- | --- | --- | --- | --- |
|  |  |  | <=2.0 folds | >2.0 folds | *P* |
| Age (y) | <55 | 50 | 31 (62.0%) | 19 (38.0%) | 0.892 |
|  | >=55 | 26 | 17 (65.4%) | 9 (34.6%) |  |
| Grade | 1&2 | 26 | 14 (53.8%) | 12 (46.2%) | 0.160 |
|  | 3 | 31 | 21 (67.7%) | 10 (32.3%) |  |
| Stage | Early | 25 | 15 (60.0%) | 10 (40.0%) | 0.373 |
|  | Late | 49 | 34 (69.4%) | 15 (30.6%) |  |
| Cell types | Endometrioid | 22 | 16 (72.7%) | 6 (27.3%) | 0.280 |
|  | Others | 54 | 33 (61.1%) | 21 (38.9%) |  |
| Cell types | Mucinous | 18 | 7 (38.9%) | 11 (61.1%) | **0.012** |
|  | Others | 58 | 41 (70.7%) | 17 (29.3%) |  |
| Cell types | Serous | 25 | 16 (64.0%) | 9 (34.0%) | 0.929 |
|  | Others | 51 | 32 (62.8%) | 19 (37.2%) |  |
| Cell types | Clear cell | 16 | 10 (62.5%) | 6 (37.5%) | 0.891 |
|  | Others | 60 | 37 (61.7%) | 23 (38.3%) |  |
| Recurrence | + | 39 | 22 (56.4%) | 17 (43.6%) | 0.130 |
|  | - | 33 | 24 (72.7%) | 9 (27.3%) |  |

**Table S5**

**AMPK-γ1 expression and clinicopathologic factors in ovarian cancer determined by Q-PCR**

| Characteristics | | Total | Expression | |  |
| --- | --- | --- | --- | --- | --- |
|  |  |  | <=2.0 folds | >2.0 folds | *P* |
| Age (y) | <55 | 50 | 33 (66.0%) | 17 (34.0%) | 0.095 |
|  | >=55 | 26 | 12 (46.2%) | 14 (53.8%) |  |
| Grade | 1&2 | 26 | 11 (42.3%) | 15 (57.7%) | 0.236 |
|  | 3 | 31 | 18 (58.1%) | 13 (41.9%) |  |
| Stage | Early | 25 | 16 (64.0%) | 9 (36.0%) | 0.570 |
|  | Late | 49 | 28 (57.1%) | 21 (42.9%) |  |
| Cell types | Endometrioid | 22 | 14 (63.6%) | 8 (36.4%) | 0.616 |
|  | Others | 54 | 31 (57.4%) | 23 (42.6%) |  |
| Cell types | Mucinous | 18 | 8 (44.4%) | 10 (55.6%) | 0.145 |
|  | Others | 58 | 37 (63.8%) | 21 (36.2%) |  |
| Cell types | Serous | 25 | 11 (44.0%) | 14 (56.0%) | **0.059** |
|  | Others | 51 | 34 (66.7%) | 17 (33.3%) |  |
| Cell types | Clear cell | 16 | 10 (62.5%) | 6 (37.5%) | 0.971 |
|  | Others | 60 | 35 (58.3%) | 25 (41.7%) |  |
| Recurrence | + | 39 | 25 (64.1%) | 14 (35.9%) | 0.389 |
|  | - | 33 | 19 (57.6%) | 14 (42.4%) |  |

**Table S6**

**AMPK-γ2 expression and clinicopathologic factors in ovarian cancer determined by Q-PCR**

| Characteristics | | Total | Expression | |  |
| --- | --- | --- | --- | --- | --- |
|  |  |  | <=2.0 folds | >2.0 folds | *P* |
| Age (y) | <55 | 50 | 33 (66.0%) | 17 (34.0%) | 0.476 |
|  | >=55 | 26 | 15 (57.7%) | 11 (42.3%) |  |
| Grade | 1&2 | 26 | 10 (38.5%) | 16 (61.5%) | **0.006** |
|  | 3 | 31 | 23 (74.2%) | 8 (25.8%) |  |
| Stage | Early | 25 | 20 (80.0%) | 5 (20.0%) | **0.024** |
|  | Late | 49 | 26 (53.1%) | 23 (46.9%) |  |
| Cell types | Endometrioid | 22 | 13 (59.1%) | 9 (40.9%) | 0.639 |
|  | Others | 54 | 35 (64.8%) | 19 (35.2%) |  |
| Cell types | Mucinous | 18 | 9 (50.0%) | 9 (50.0%) | 0.185 |
|  | Others | 58 | 39 (67.2%) | 19 (32.8%) |  |
| Cell types | Serous | 25 | 9 (36.0%) | 16 (64.0%) | **0.001** |
|  | Others | 51 | 39 (76.5%) | 12 (23.5%) |  |
| Cell types | Clear cell | 16 | 16 (100.0%) | 0 (0.0%) | **<0.001** |
|  | Others | 60 | 32 (53.3%) | 28 (46.7%) |  |
| Recurrence | + | 39 | 24 (61.5%) | 15 (38.5%) | 0.530 |
|  | - | 33 | 22 (66.7%) | 11 (33.3%) |  |
